# Patterns of microbial resistance in bloodstream infections of hemodialysis patients: a cross-sectional study from Palestine

**DOI:** 10.1038/s41598-022-21979-7

**Published:** 2022-10-26

**Authors:** Shatha A. AbuTaha, Tasbeeh Al-Kharraz, Souad Belkebir, Adham Abu Taha, Sa’ed H. Zyoud

**Affiliations:** 1 Department of Medicine, College of Medicine and Health Sciences, An-Najah National University, Nablus, 44839 Palestine; 2 Family and Community Medicine Department, Faculty of Medicine and Health Sciences, An-Najah National University, Nablus, 44839 Palestine; 3 Department of Biomedical Sciences, Faculty of Medicine and Health Sciences, An-Najah National University, Nablus, 44839 Palestine; 4 Department of Pathology, An-Najah National University Hospital, Nablus, 44839 Palestine; 5 Department of Clinical and Community Pharmacy, College of Medicine and Health Sciences, An-Najah National University, Nablus, 44839 Palestine; 6 Poison Control and Drug Information Center (PCDIC), College of Medicine and Health Sciences, An-Najah National University, Nablus, 44839 Palestine; 7 Clinical Research Center, An-Najah National University Hospital, Nablus, 44839 Palestine

**Keywords:** Microbiology, Nephrology

## Abstract

Bloodstream infections (BSIs) are a prominent cause of death and hospitalization among hemodialysis (HD) patients. The emergence of multidrug-resistant organisms (MDRO) is making the management of these infections more challenging. This study describes the clinical characteristics, microbial profiles and antibiotic resistance patterns in patients with BSIs. A retrospective cross-sectional study was conducted at An-Najah National University Hospital from January 2019 to December 2020. Clinical and demographic data regarding BSIs were collected from the hospital information system. Data regarding bacterial isolates and the antimicrobial resistance of BSIs were collected from the microbiology lab. Data were entered and analyzed using version 21 of the Statistical Package for Social Sciences program (IBM-SPSS). 111 BSIs occurred during the study period, with a rate of 1.5 infections per 100 patient-months. These patients had been on HD for the median duration of 747 (360, 1825) days and 62.2% had already had a BSI before the study period. 118 microorganisms were isolated; 99 (83.89%) were gram-positive and 19 (16.1%) were gram-negative. Among the gram-positive isolates, coagulase-negative staphylococci (CoNS) (88, 74.57%) were predominant. As for the gram-negative isolates, the most frequent were both *Stenotrophomonas maltophilia* and *Escherichia coli*, with five (4.23%) positive cultures each. Among the latter, two were Extended-Spectrum Beta-Lactamase producing (ESBL) (1.69%). The most frequently used empiric antibiotics were a combination of vancomycin and gentamicin (27%), followed by vancomycin alone (24.3%). Regarding gram-positive isolates, vancomycin was the most frequently used and effective antibiotic after cultures, whereas for gram-negative bacteria, it was found to be gentamicin. MDROs were defined as those resistant to at least one agent in three or more antimicrobial categories. 89 (75.4%) isolates were found to be MDRO, 85 (85.85%) gram-positive bacteria and 4 (21%) gram-negative bacteria. When comparing patients according to the type of vascular access, 66 (75%) infections with MDRO were found among patients with central venous catheters (CVCs). However, no statistically significant relationship was found between the type of vascular access and infection with MDRO (*p* = 0.523). MDRO cause a remarkably high proportion of BSIs in Palestinian patients. The results of this study support the empiric use of vancomycin and gentamicin to treat these infections. It is vital that health care providers prevent these infections via instituting and adhering to infection control policies in hemodialysis centers and providing proper antibiotic therapy of limited use and duration when necessary to avoid breeding resistance.

## Introduction

Chronic Kidney Disease (CKD) is the presence of kidney damage or an estimated glomerular filtration rate (eGFR) less than 60 ml/min/1.73 m^2^, persisting for 3 months or more, irrespective of the cause^1^. It is classified into six categories based on glomerular filtration rate, in which stage G5 [End Stage Renal Disease (ESRD)] is the final and most serious stage. Among these patients, infections are some of the most noteworthy complications, since they are the second most common cause of hospitalization after cardiovascular disease^2^. Furthermore, they are a leading cause of death in hemodialysis patients, second only to cardiovascular disease, and accounting for ~ 35% of all-cause mortality^3^.

The National Healthcare Safety Network (NHSN) Dialysis Event Surveillance Protocol defines a bloodstream infection (BSI) as a positive blood culture collected from a hemodialysis patient as an outpatient or within 1 calendar day after hospital admission^4^. These are the most frequently found infections among hemodialysis patients, and are associated with serious morbidity and mortality^5^.

BSI may also lead to a number of metastatic complications, including infective endocarditis, vertebral osteomyelitis, spinal epidural abscess, and septic arthritis^6^. In addition, infections, both local and systemic, are the most common chronic complications when working with tunnelled hemodialysis catheters^7^. These complications are devastating and are often associated with poor outcomes. Additionally, BSIs incur significant costs; about $21,000–$24,000 per episode in the US^8^, and a total of 49.01 million CDN per year in Canada^9^.

When compared to the general population, hemodialysis patients have higher rates of infectious diseases, particularly sepsis, and associated mortality^10^. This is due to a variety of reasons, including advanced age, impaired immunity due to renal failure, anemia, the hemodialysis procedure itself and vascular access, comorbid conditions and malnourishment^11^.

The rates of BSIs found in hemodialysis patients differ according to the type of venous access used. The risk of infection has been found to be higher among patients using central venous catheters (CVC), when compared to those who use arteriovenous fistulas (AVF) or arteriovenous grafts (AVG)^12–15^. However, due to anatomic host factors, patient reluctance, and prolonged maturation time, it is not always possible to use an AVF, and CVC usage is often necessary, especially in incident cases^16^. In Palestine, AVFs are the most common access type, followed by catheters and, finally AVGs. CVC usage is more common among incident patients, while AVFs are more common in prevalent patients^17^.

In addition to the use of CVCs in hemodialysis, a history of bacteremia, hypoalbuminemia, malnutrition, anemia, diabetes mellitus, and colonization by methicillin-resistant *Staphylococcus aureus* (MRSA) are risk factors for the development of BSI in hemodialysis patients^18–20^. Notably, a number of these risk factors are known to exist to a significant extent among Palestinian hemodialysis patients; one study found that most (65%) of these patients were moderately malnourished^21^, while another found that diabetes mellitus was the most common cause of CKD among patients in a certain district^22^.

The most commonly isolated bacteria in blood cultures from hemodialysis patients with BSI are gram-positive bacteria, mainly *Staphylococcus aureus* and coagulase-negative staphylococci^18,23,24^. These organisms are often found to exhibit antibiotic resistance, due to recurrent hospital admissions and the recurring need for antimicrobial therapy^24^. Notably, isolation of *S. aureus*, and isolation of a resistant microorganism, in addition to patient age, are independent risk factors for morbidity and mortality in this patient population^25^.

Antibiotic resistance is a growing threat with potentially grave public health consequences. Factors like improper prescription practices and lack of appropriate functioning drug regulatory mechanisms contribute to this increasing risk and are especially prominent in developing countries^26^. Thus far, there have been no published studies showing the epidemiology and patterns of antibiotic resistance of etiological agents in Palestinian hemodialysis patients.

This study provides information regarding the demographic and clinical characteristics of hemodialysis patients with BSIs at An-Najah National University Hospital. It also investigates the spectrum of bacterial isolates and their antibiotic resistance patterns. The information provided will aid in decreasing morbidity and mortality among this patient population by aiding in the establishment, revision, and modification of empirical treatment guidelines based on the latest data. It will inform healthcare providers of the clinical characteristics associated with BSIs and will drive quality improvement initiatives to lower therapeutic health care costs and improve patient outcomes.

## Methods

### Study design

A retrospective cross-sectional study was conducted to determine the demographic and clinical characteristics of hemodialysis patients with BSIs, in addition to the frequency of bacterial isolates and patterns of bacterial resistance.

### Study setting

The study was conducted at An-Najah National University Hospital in Nablus, Palestine. It is a teaching hospital and the main hemodialysis center in the northern West Bank. It offers hemodialysis services to a total population of more than 300,000 people living in Nablus district.

### Study population

All ESRD patients who underwent hemodialysis at An-Najah National University Hospital in the period of time between January 2019 and December 2020.

### Data collection

Demographic and clinical data regarding BSIs were obtained from the hospital information system (HIS). Information regarding the specimens, types of microorganisms, and antibiotic susceptibility of BSIs was collected from the microbiology lab. For each positive blood culture to be considered a separate hemodialysis event, there were 21 or more days between positive blood cultures, as recommended by the NHSN Dialysis Event Surveillance Protocol^27^. If the organisms were different, it was still not considered a new dialysis event, but the new organisms were added to the first reported event.

### Microorganism identification and drug-susceptibility testing

Blood samples were inoculated into VersaTrek Redox 1 aerobic and Redox 2 anaerobic media (Thermo Fisher Scientific, Waltham, MA) and evaluated using the VersaTREKTM automated microbial detection system (TREK Diagnostic Systems, Cleveland, OH, USA). Incubation was continued for up to 5 days or until a positive culture was observed. Positive bacterial cultures were examined for pathogen type and antibiotic sensitivity. VITEK 2^®^ COMPACT (bioMérieux, Marcy-I'Étoile, France) was used to identify bacteria and their antibiotic sensitivity. GN and GP cards were used to identify gram-negative and gram-positive microorganisms, respectively. AST-GN204 and AST-GN222 labels were used to test antibiotic sensitivity in gram-negative bacteria. AST-GP67 and AST-GP 03 cards were used to determine gram-positive sensitivity. The VITEK 2^®^ COMPACT was used to determine multiresistance phenotypically from the antibiotic susceptibility profile. The Clinical Laboratory Standards Institute standard was used to interpret drug susceptibility data (CLSI, United States). MDROs were defined as those resistant to at least one agent in three or more antimicrobial categories.

### Inclusion criteria

All patients with ESRD who underwent hemodialysis at An-Najah National University Hospital during the study period and had positive blood cultures were included.

### Exclusion criteria

Patients who did not have positive blood cultures, patients on peritoneal dialysis, and patients under 18 years of age were excluded.

### Statistical analysis

Data were entered and analyzed using version 21 of the Statistical Package for Social Sciences program (IBM Corp., Armonk, NY, USA). For continuous variables, data were expressed as means ± SD or as median and interquartile ranges for variables which were not normally distributed. Categorical variables were expressed as frequencies and percentages. Comparisons between groups according to vascular access type were performed via an independent sample t-test for continuous variables that were normally distributed and Mann–Whitney test for continuous variables that were not normally distributed. For categorical variables, Chi-square test was performed to determine the relationship between the type of vascular access and history of BSI and Fisher’s exact test was performed for the rest. Level of significance was a P value ≤ 0.05.

### Ethics approval and consent to participate

The proposal was reviewed and accepted by the research committee of An-Najah National University on October 11th, 2020. Approval of *Institutional Review Boards (IRB) of An-Najah National University* was obtained on October 13th, 2020. P All the methods used in the study were carried out in accordance with the relevant guidelines and regulations. Permission was obtained from An-Najah National University Hospital to access patient files. All hemodialysis patients gave written informed consent to use their health records for analysis in any scientific research.

## Results

### Demographic and clinical characteristics of the study population

A total of 111 cases of BSI occurred during the study period, with an incidence rate of 1.5 infections per 100 patient-months. Of these, 71 (64%) were male and 40 (36%) were female. The mean age ± SD of the study participants was 58.15 ± 16.6, ranging from 18 to 88 years. 66 (59.5%) patients had diabetes mellitus and 99 (89.2%) had hypertension. These patients had been on hemodialysis for a median (IQR) duration of 747 (360, 1825) days. Among these patients, 69 (62.2%) had already had a previous BSI before the study period.

Many patients had more than one BSI during the study period, with a mean ± SD of 1.8 ± 1.025 episodes occurring in each patient. 37 (33.3%) had two episodes, 13 (11.7%) had three episodes, two (1.8%) had four episodes and five (4.5%) had five episodes. The suspected source of infection was vascular access in 107 (96.4%) patients, and was determined to be uncertain in the remaining four (3.6%). Seven (6.3%) patients were hospitalized due to their BSI, one (0.9%) had endocarditis as a complication, and three (2.7%) died during their hospital admission (Table 1).

**Table 1:** Demographic and clinical characteristics of HD patients who had BSI (111 patients).

Variable	n (%)
Age (years), mean ± SD	58.15 ± 16.6
**Gender**	
Male	71 (64)
Female	40 (36)
**Diabetes mellitus**	
No	45 (40.5)
Yes	66 (59.5)
**Hypertension**	
No	12 (10.8)
Yes	99 (89.2)
Duration of HD (days), median and IQR	747 (360–1825)
**Previous history of BSI**	
No	41 (36.9)
Yes	69 (62.2)
**Suspected source of infection**	
Vascular access	107 (96.4)
Uncertain	4 (3.6)
Other or contamination	0
**Hospitalization**	
No	104 (93.7)
Yes	7 (6.3)
Number of BSI during study period, mean ± SD	1.8 ± 1.025
**Complications**	
None	110 (99.1)
Endocarditis	1 (0.9)
Osteomyelitis and others	0
**Outcome**	
Recovered	108 (97.3)
Died	3 (2.7)
Total	111 (100)

*BSI* bloodstream infection, *HD* hemodialysis, *IQR* interquartile range, *SD* standard deviation.

The most common means of vascular access among these patients was via CVC in 82 (73.9%) patients. Patients who were using a CVC for dialysis whilst their AVF was maturing were counted among the CVC group as well. Among patients with CVCs, the most common location was the right internal jugular vein (38, 34.2%), then both the left internal jugular vein and the left femoral vein (16, 14.4%). These were followed by the right femoral vein (10, 9%), and the subclavian vein (2, 1.8%). These patients had had their CVC in place for a median (IQR) duration of 90 (33, 259) days, and 32 (39%) patients had their CVCs removed following the occurrence of their BSI.

26 (23.4%) patients had AVFs and 2 (1.8%) patients had AVGs, one of which was left femoral (1, 0.9%) and the other was right brachioaxillary (1, 0.9%). AVFs were most commonly left brachiocephalic (21, 18.9%), followed by right brachiocephalic (5, 4.5%). Vascular access details of HD patients who had BSI are shown in Additional file 1: Table S1.

When comparing the patients according to their vascular access type, we found that patients with CVCs had a greater number of BSIs during the study period [82 vs. 28], and a higher percentage of previous BSIs [(68.3%) vs. (48.1%)] when compared to patients with AVFs or AVGs. They also had a higher percentage of hospitalizations [(8.5%) vs. (0%)], and a higher percentage of deaths [(3.7%) vs. (0%)]. However, none of these relationships were deemed statistically significant. (*P* = 0.053, 0.06, 0.188 and 0.569, respectfully); (Table 2).

**Table 2:** BSI features by vascular access type (merged AVF and AVG).

Variables	CVC	AVF/AVG	P value (CVC vs. AVF/AVG)
	n (%)	n (%)	
**Previous history of BSI**			0.060^a^
No	26 (31.7)	14 (51.9)	
Yes	56 (68.3)	13 (48.1)	
**Number of BSI during study period**			0.053^b^
One	36 (43.9)	17 (60.7)	
Two	32 (39)	5 (17.9)	
Three	7 (8.5)	6 (21.4)	
Four	2 (2.4)	0	
Five	5 (6.1)	0	
**Suspected source of infection**			0.569^**b**^
Vascular access	79 (96.3)	28 (100)	
Uncertain	3 (3.7)	0	
**Hospitalization**			0.188 ^**b**^
No	75 (91.5)	28 (100)	
Yes	7 (8.5)	0	
**Outcome**			0.569 ^**b**^
Recovered	79 (96.3)	28 (100)	
Died	3 (3.7)	0	
Total (110)	82 (100)	28 (100)	

*CVC* central venous catheter, *AVF* arteriovenous fistula, *AVG* arteriovenous graft, *BSI* bloodstream infection.

^a^Chi-square test.

^b^Fisher’s exact test.

These patients had a mean ± SD hemoglobin of 9.84 ± 1.57, a median (IQR) albumin of 3.6 (3.1–3.91), and a median (IQR) ferritin of 634 (293–915). Four (3.6%) patients in this study had hepatitis C, and one (0.9%) patient had hepatitis B, as shown in Additional file 1: Table S2. No statistically significant relationship was found between these lab values and the type of vascular access. Laboratory characteristics by vascular access type are shown in Additional file 1:Table S3.

### Microbial profiles

Among the 111 positive blood cultures, 118 microorganisms were isolated. 99 (83.89%) isolates were gram-positive and 19 (16.1%) were gram-negative. No fungal infections were found during the study period.

Among the gram-positive isolates, coagulase-negative staphylococci (CoNS) (88, 74.57%) were predominant, followed by methicillin-sensitive *Staphylococcus aureus* (5, 4.23%), and *Bacillus *spp*.* (3, 2.54%). These were followed by methicillin-resistant *Staphylococcus aureus* (MRSA), *Enterococcus faecalis,* and *Streptococcus salivarius* with one (0.84%) positive culture.

As for the gram-negative isolates, the most frequent were both *Escherichia coli* (*E. coli*) and *Stenotrophomonas maltophilia*, with five (4.23%) positive cultures each. *E. coli* can be divided into non-Extended-Spectrum Beta-Lactamase producing *Escherichia coli* (*E. coli* non-ESBL) (3, 2.54%) and ESBL-producing *Escherichia coli* (2, 1.69%). These were followed *by Enterobacter cloacae* (3, 2.54%), then both *Cedecea lapagei* and *Klebsiella pneumonia* with two (1.69%) positive cultures each, and finally *Pseudomonas aeruginosa* and *Rosemonas gilardii* with one (0.84%) positive blood culture each. (Table 3). When comparing patients based on the type of vascular access, no statistically significant relationship was found between the type of vascular access and the type of microorganism (*p* = 0.812 using Fisher’s exact test). Microbial isolates by vascular access type are shown in Additional file 1:Table S4.

**Table 3:** Frequency and percentage of isolated microorganisms.

Microorganism	Frequency
Total number 118	N (%)
**Gram-negative, total**	**19 (16.1)**
*E. coli non ESBL*	3 (2.54)
*E. coli ESBL*	2 (1.69)
*Klebsiella pneumoniae*	2 (1.69)
*Pseudomonas aeruginosa*	1 (0.84)
*Stenotrophomonas maltophilia*	5 (4.23)
*Rosemonas gilardii*	1 (0.84)
*Enterobacter cloacae*	3 (2.54)
*Cedecea lapagei*	2 (1.69)
**Gram-positive, total**	**99 (83.89)**
*CoNS*	88 (74.57)
*Staphylococcus aureus*	5 (4.23)
*MRSA*	1 (0.84)
*Enterococcus faecalis*	1 (0.84)
*Streptococcus salivarius*	1 (0.84)
*Bacillus spp.*	3 (2.54)

*ESBL* extended spectrum beta-lactamase, *CoNS* coagulase-negative staphylococci, *MRSA* methicillin-sensitive *Staphylococcus aureus.*

### Antimicrobials used before and after culture

A combination of vancomycin and gentamicin was the most popular empiric therapy, utilized in 30 (27%) cases, followed by vancomycin alone (27, 24.3%), vancomycin and amikacin (11, 9.9%), gentamicin alone (5, 4.5%) and a combination of vancomycin and ceftriaxone (4, 3.6%). Other less common options included amikacin alone, a combination of vancomycin, gentamicin, and meropenem, and a combination of vancomycin, gentamicin, and amikacin, each of which was used twice (1.8%). Finally, the combination of vancomycin and meropenem was used only once (0.9%) (Table 4).

**Table 4:** Frequency and percentages of empiric and culture-guided antimicrobials used.

	n (%)
**Empiric antimicrobials**	
Vancomycin	27 (24.3)
Gentamicin	5 (4.5)
Amikacin	2 (1.8)
Vancomycin + Gentamicin	30 (27)
Vancomycin + Amikacin	11 (9.9)
Vancomycin + Ceftriaxone	4 (3.6)
Vancomycin + Gentamicin + Meropenem	2 (1.8)
Vancomycin + Gentamicin + Amikacin	2 (1.8)
Vancomycin + Meropenem	1 (0.9)
Missing	27 (24.3)
**Culture-guided antimicrobials**	
Vancomycin	39 (35.1)
Gentamicin	5 (4.5)
Amikacin	2 (1.8)
Vancomycin + Gentamicin	23 (20.7)
Vancomycin + Amikacin	9 (8.1)
Vancomycin + Ceftriaxone	3 (2.7)
Vancomycin + Gentamicin + Meropenem	2 (1.8)
Vancomycin + Gentamicin + Amikacin	1 (0.9)
Missing	27 (24.3)

As for culture-guided antibiotics, vancomycin was the most frequently used (39, 35.1%), followed by a combination of vancomycin and gentamicin (23, 20.7%), a combination of vancomycin and amikacin (9, 8.1%) and gentamicin alone (2, 1.8%). Less common choices included a combination of vancomycin and ceftriaxone (3, 2.7%), amikacin alone (2, 1.8%), and a combination of vancomycin, gentamicin and meropenem (2, 1.8%). Finally, the combination of vancomycin and meropenem was used only once (0.9%) (Table 4).

### Antimicrobial resistance of gram-positive bacterial isolates

Among the coagulase-negative staphylococci (CoNS) included in our study, 96.5% were resistant to benzylpenicillin, 88.6% were resistant to oxacillin, and 88.5% were resistant to amoxicillin/clavulanic acid. Similarly, high resistance rates were also found against cefuroxime (89.5%), erythromycin (77%), ciprofloxacin (70.4%) and levofloxacin (69%). Meanwhile, lower resistance rates were found against other antimicrobials such as moxifloxacin (39.7%), clindamycin (42.5%), trimethoprim/sulfamethoxazole (TMP/SMX) (36%), gentamicin (35%), tetracycline (18%) and rifampicin (4.5%). None of the CoNS isolated were resistant to vancomycin, linezolid, doxycycline, tigecycline, piperacillin/tazobactam, or quinupristin/dalfopristin.

As for *Staphylococcus aureus*, one isolate was found to be methicillin-resistant (MRSA), while 5 isolates were methicillin-sensitive. Among the latter group, 100% were resistant to benzylpenicillin, while resistance to both clindamycin and erythromycin was found to be 80%.

Among the 3 isolates of *Bacillus* spp., 100% were resistant to clindamycin and ceftriaxone, 66.6% were resistant to amoxicillin/clavulanic acid and ceftazidime, 50% were resistant to meropenem and piperacillin/tazobactam and 33% were resistant to amikacin. Only one isolate of *Enterococcus faecalis* was found during the period of the study, and it was found to be resistant to gentamicin, ciprofloxacin, quinupristin/dalfopristin and erythromycin. Rates of antibiotic resistance among the most common gram-positive bacterial isolates are shown in Additional file 1:Table S5.

### Antimicrobial resistance of gram-negative bacterial isolates

Among the most frequently isolated gram-negative bacteria, *Steotrophomonas maltophilia*, 75% were resistant to ceftazidime, and 20% were resistant to TMP/SMX, while all isolates were sensitive to cefepime. As for *E. coli*, 2 isolates were found to be extended spectrum beta-lactamase-producing (ESBL), while 3 isolates were not. Among the latter group, 66.6% were resistant to cefepime and cefotaxime, 50% were resistant to TMP/SMX and ceftazidime, and 33.3% were resistant to amoxicillin/clavulanic acid.

Two isolates of *Cedecea lapagei* and *Klebsiella pneumonia* were found during the study period; of the former, 100% were resistant to tetracycline, and of the latter, 50% were resistant to ceftazidime, cefotaxime and ceftriaxone, while 100% were resistant to ampicillin, amoxicillin/clavulanic acid, ciprofloxacin, gentamicin, and TMP/SMX. As for *Pseudomonas aeruginosa* and *Rosemonas gilardi*, one positive culture of each were found during the study period; the former was resistant to imipenem, and the latter was resistant to cefotaxime, TMP/SMX and ceftazidime. Rates of antibiotic resistance among the most common gram-negative bacterial isolates are shown in Additional file 1:Table S5.

### Multidrug-resistant organisms

Multidrug resistance is an acquired non-susceptibility to at least one agent in three or more antimicrobial categories^28^. A total of 89 (75.4%) bacterial isolates were found to be multidrug-resistant organisms (MDRO), 85 (85.85%) gram-positive bacteria and 4 (21%) gram-negative bacteria. Upon comparison of patients according to the type of vascular access, 66 (75%) infections with MDRO were found among patients with CVCs, while 22 (25%) were found among patients with AVFs or AVGs. However, no statistically significant relationship was found between the type of vascular access and infection with MDRO (*p* = 0.523 using Chi square test) (Table 5).

**Table 5:** Frequency and percentage of MDRO by vascular access type (merged AVF and AVG).

	CVC	AVF/AVG
Not MDRO—n (%)	20 (69)	9 (31)
MDRO—n (%)	66 (75)	22 (25)

*MDRO* multidrug-resistant organisms, *CVC* central venous catheter, *AVF* arteriovenous fistula, *AVG* arteriovenous graft.

## Discussion

Infections are a prominent cause of morbidity and mortality in patients with ESRD, and are the second leading cause of death after cardiovascular disease^3^. The United States Renal Data System (USRDS) in its 2020 data report revealed that, while adjusted rates of hospitalization for cardiovascular and other causes decreased over time, the rate of infection-related hospitalizations remained relatively unchanged between 2009 and 2018^2^. Furthermore, it has been found that the rates of bloodstream infections (BSIs), the most common infections among hemodialysis patients, have increased by 40% between 2003 and 2014^5^.

The rate of BSI in this study was 1.5 infections per 100 patient-months. This was quite higher than the rate seen in a study in Saudi Arabia, which was 0.4 per 100 patient months^29^, and a study in Greece, which was 0.52 per 1000 patient-days^18^.

The type of vascular access is an established risk factor for BSI among hemodialysis patients. It has been previously shown that, when contrasted against patients who were using an AVF, patients with CVCs experienced a 20-times higher risk of access-related bacteraemia^23^. In our study, 82 (74%) BSI episodes occurred in patients with CVCs, while 26 (23.4%) had an AVF and 2 (1.8%) had an AVG. These results are similar to those found in the aforementioned Greek study, where 84% of episodes occurred in patients with CVCs^18^. In the Saudi study, all episodes were found in patients with CVCs^29^. Another study showed that the rate of access-related BSIs in patients with CVCs was significantly higher than in patients with AVFs, with rates of 2.22 per 100 patient-months, vs. 0.11 per 100 patient-months, respectively (*p* < 0.001)^23^. It has also been shown that the type of CVC plays a role in the incidence of BSI as well, with more cases found among patients with temporary catheters when compared to permanent catheters^18,30^.

Regarding catheter location, 48.6% of patients in our study had internal jugular venous catheters, while 23.4% had femoral venous catheters. It has been previously shown that, in the acute hemodialysis setting, the site of the highest infection rate correlates with body habitus; in patients with lower body mass indexes (BMIs) the risk is greatest at the jugular site, while in patients with BMIs > 28.2, the risk is greater at the femoral site^31^.

Among patients with CVCs in our study, 68.3% had a history of BSI before the study period, 8.5% were hospitalized, and 3.7% died during their hospital admission. These patients also had a greater number of recurrent infections, with 56% having two or more infections during the study period. Additionally, 75% of infections with MDRO were found in patients with CVCs. Hemodialysis via CVC, compared with that via AVF or AVG, has been shown to increase not only the rate of infection, but also mortality^32,33^. However, in our study no significant difference in mortality was found between different vascular access types, presumably due to the small number of deaths.

Gram-positive bacteria were the cause of the majority of BSIs in the present study (83.89%), with the two most common isolated pathogens being CoNS (74.57%) and *Staphylococcus aureus* (5.07%), one of which was MRSA. These findings are consistent with those of various other investigators^18,23,30,31^. Among gram-negative bacteria, the most common isolates were both *Stenotrophomonas maltophilia* and *E. coli* (4.23%), followed by *Enterobacter cloacae* (2.54%). Although in our study *Klebsiella pneumoniae* accounted for only 1.69% of infections, in other studies^29,34^ it accounted for the majority of infections, both overall and among gram-negative bacteria.

Resistant organisms are often the cause of infections in hemodialysis patients, due to their frequent contact with health care facilities and hospitalizations, temporary catheters used for dialysis, and frequent need for antimicrobial therapy^35^. In our study, we found that 62.2% of patients had had a previous BSI before, and thus had a history of prior antimicrobial use. Fram et al. found that previous antimicrobial use was significantly associated with a higher occurrence of BSI^15^. In this study, 75.4% of bacterial isolates were MDRO. Comparatively, in the aforementioned Saudi study, multiply resistant bacteria were a little over a third^29^. Our remarkably high proportion of MDRO reflects the problem of irrational antibiotic usage in our community.

Among the most common pathogens in our study, CoNS and *Staphylococcus aureus*, 88.6% of the former and 83.3% of the latter were found to be MDRO. These findings are in concordance with what has been published in previous literature. Worldwide, over 95% of nosocomial CoNS BSIs are resistant to methicillin, and dialysis patients constitute up to 15% of all invasive MRSA infections^31^. A Greek study found that over two-thirds of *Staphylococcus aureus* and CoNS were methicillin resistant^18^, while a Brazilian study found that, among gram-positive cocci, 72.7% of *Staphylococcus aureus* and 100% of CoNS were methicillin resistant^30^. Only one isolate of MRSA in our study was found to be vancomycin-resistant, and thus local patterns of resistance support the empiric use of vancomycin as a first-line therapy.

While gram-positive cocci preponderate as a cause of BSI in hemodialysis patients, recently the proportion of BSIs caused by gram-negative pathogens is increasing, with one study showing a statistically significant increase in gram-negative pathogens and a decrease in *Staphylococcus aureus* causing BSIs over a 9-year period^5^. Another study showed that a quarter of BSIs were due to gram-negative pathogens resistant to common antimicrobials, with 25% of these isolates only sensitive to colistin^18^. Twenty-one percent of gram-negative isolates in this study were found to be MDRO, among which were two isolates of *Klebsiella pneumoniae*, and two isolates of *ESBL*-producing *E. coli*. Both *Klebsiella pneumoniae* isolates exhibited resistance against gentamicin, ciprofloxacin, ampicillin, amoxicillin/clavulanic acid, and TMP/SMX, with one of the isolates additionally exhibiting resistance against 3rd generation cephalosporins. In the aforementioned Saudi study, where *Klebsiella pneumoniae* was the most frequently isolated bacteria, all isolates were resistant to ampicillin. This is comparative to a study in Algeria^34^, where all isolated *Klebsiella pneumoniae* strains were ESBL-producing and resistant to at least gentamicin.

As for ESBL-producing *E. coli*, both isolates in our study exhibited resistance against 3rd generation cephalosporins, ciprofloxacin, ampicillin, and TMP/SMX, with one isolate additionally resistant to gentamicin and amoxicillin/ clavulanic acid. *E. coli* resistance to 3rd generation cephalosporins was also seen in the aforementioned Brazilian study^30^.

BSI prevention is essential to decrease antibiotic usage and subsequently decrease the development of antibiotic resistance. Adherence to hand hygiene protocols, proper catheter insertion and handling, and in some patients, catheter-lock solutions have been proven to be of benefit in the reduction of BSI risk^31^. Antibiotic stewardship programs are essential, as it has been shown that the implementation of antibiotic stewardship programs is associated with a decline in antimicrobial prescribing with no negative effects^36^. Antibiotic treatment should be individualized according to bacterial susceptibility profiles, keeping in mind the different pharmacokinetics of frequently used antimicrobials when compared with patients with normal kidney function, and the risk of developing resistance at the level of the individual on one hand, and the community on the other.

In our study, the most frequent empiric antibiotic therapy was a combination of vancomycin and gentamicin (27%), followed by vancomycin alone (24.3%). Culture-guided antibiotic choices were similar, with the most frequent therapy being vancomycin alone (35.1%), followed by a combination of vancomycin and gentamicin (20.7%). In general, vancomycin is the most frequently used systemic antibiotic in patients on hemodialysis^31^. In the aforementioned Saudi study, vancomycin was used in most BSI cases, followed by ceftazidime and gentamicin, with no resistance against either vancomycin or ceftazidime was found in that study^29^. Similarly, an Algerian study found that 3rd generation cephalosporins and vancomycin were most commonly used as empiric therapy^34^. Notably, they found that deaths that occurred during their study period occurred when empiric therapy failed to target gram-negative bacteria.

Fitzgibbons et al. recommended cefazolin as an appropriate first-line agent to treat BSIs caused by methicillin-sensitive CoNS and *S. aureus*, and vancomycin to treat their methicillin-resistant counterparts^31^. Additionally, aminoglycoside synergy with beta-lactam or glycopeptide antibiotics has been shown to decrease the duration of bacteremia in hemodialysis patients, in addition to providing coverage for suspected gram-negative infections. Vancomycin is also first-line with regard to treating vancomycin-sensitive *E. faecium*, while daptomycin is recommended in the case of vancomycin resistance. As for *E. faecalis* bacteremia, ampicillin with aminoglycoside synergy is the preferred treatment.

Concerning CVC removal, hemodialysis patients represent a singular challenge, as their continuous need for vascular access complicates the removal of catheters after infection. In our study, catheters were removed following infection in 32 (39%) patients. The Infectious Disease Society of America provides evidence-based guidelines on the matter, allowing catheter salvage in organism-and-situation-specific circumstances^37^. For example, while eradication of BSIs caused by *S. aureus* requires removal of the catheter, using a combination of systemic antibiotics and antibiotic lock therapy is sufficient and allows catheter retention in uncomplicated BSIs caused by CoNS. In general, catheters should be removed in the presence of hemodynamic instability, difficult to clear organisms like *S. aureus*, *Pseudomonas* or fungi, severe sepsis, complications such as endocarditis or metastatic infection, recurrence of uncomplicated central line-associated bloodstream infections, and bacteremia that persists beyond 72 h of appropriate antibiotic treatment^38^.

This paper is the first in Palestine to study the topic of BSI in hemodialysis patients. However, our study has a number of limitations. First, there were some missing data regarding the antibiotics used in the treatment of BSIs, and the whether the CVCs used in dialysis were temporary or permanent catheters. Second, this is a single-center study and may not be representative of other centers. Thirdly, this is a retrospective study, and is susceptible to the drawbacks of retrospective studies regarding overestimation or underestimation of associations due to the influence of unmeasured confounding. Finally, our sample size was relatively small, thus potentially diminishing the power of the study, and limiting the conclusions that can be drawn from it.

## Conclusions

In conclusion, BSIs remain a significant complication among hemodialysis patients in Palestine, especially among patients with CVCs, and are associated with significant morbidity, mortality, and cost. The emergence of multidrug-resistant organisms, especially MRSA and ESBL-producing bacteria, makes managing these infections even more challenging. MDRO cause a remarkably high proportion of infections in our patients. The results of this study support the empiric use of vancomycin and gentamicin to treat these infections. It is vital that health care providers prevent these infections via instituting and adhering to infection control policies in hemodialysis centers, providing proper antibiotic therapy of limited use and duration when necessary to avoid breeding resistance. The limited sample size in our study, which is a retrospective, and single-center study, is important limitation. These prevented denoting statistical significance, particularly regarding the association between CVCs and the incidence of BSIs. However, despite these negative results, we must emphasize that the existing evidence strongly supports the use of AV access rather than CVCs whenever possible.

## Supplementary Information

Supplementary Information.

## Data Availability

The data from our surveillance are not available in the public domain due to privacy and ethical restrictions, but anyone interested in using the data for scientific purposes is free to request permission from the corresponding author. Dr. Sa’ed H. Zyoud (saedzyoud@yahoo.com). Anyone requesting access to the data will receive anonymized data so that the privacy of the patients and respect for their data are maintained. This manuscript forms part of a Doctor of Medicine graduation project submitted to An-Najah National University. The abstract was published as part of self-archiving in institutional repositories (university repository: https://repository.najah.edu/handle/20.500.11888/16049).

## Abbreviations

ESRD: End-stage renal disease
BSI: Bloodstream infection
MDRO: Multidrug-resistant organisms
HD: Hemodialysis
IQR: Interquartile range
SD: Standard deviation
CKD: Chronic kidney disease
CVC: Central venous catheters
AVG: Arteriovenous grafts
AVF: Arteriovenous fistula
MRSA: Methicillin-resistant *Staphylococcus aureus*
ESBL: Extended spectrum beta-lactamase
CoNS: Coagulase-negative Staphylococci
