# Supplementary material for: Patterns of microbial resistance in bloodstream infections of hemodialysis patients: a cross-sectional study from Palestine
Source: Sci Rep. 2022 Oct 26;12:18003. doi: 10.1038/s41598-022-21979-7 (PMC9605991; doi:10.1038/s41598-022-21979-7)
Supplement: Supplementary file 1 — Supplementary Information. [file 41598_2022_21979_MOESM1_ESM.docx]

**Additional file 1: Table S1-S6. Table S1:** Vascular access details of HD Patients who had BSI. **Table S2:** Laboratory characteristics of HD patients who had BSI. **Table S3:** Laboratory characteristics by vascular access type (merged AVF and AVG). **Table S4:** Microbial isolates by vascular access type (merged AVF and AVG). **Table S5:** Rates of antibiotic resistance among the most common gram-positive bacterial isolates. **Table S6:** Rates of antibiotic resistance among the most common gram-negative bacterial isolates

**Table S1:** **Vascular access details of HD Patients who had BSI**

| **Variable** | **n (%)** |
| --- | --- |
| **Type of Access** |  |
| CVC | 82 (73.9) |
| AVF | 26 (23.4) |
| AVG | 2 (1.8) |
| **Access Location** |  |
| - **of CVC** |  |
| Left Internal Jugular Vein | 16 (14.4) |
| Right Internal Jugular Vein | 38 (34.2) |
| Left Femoral Vein | 16 (14.4) |
| Right Femoral Vein | 10 (9) |
| Left Subclavian Vein | 1 (0.9) |
| Right Subclavian Vein | 1 (0.9) |
| - **of AVF** |  |
| Left Brachiocephalic | 21 (18.9) |
| Right Brachiocephalic | 5 (4.5) |
| - **of AVG** |  |
| Left Femoral | 1 (0.9) |
| Right Brachioaxillary | 1 (0.9) |
| **CVC duration (days), median and IQR** | 90 (33-259) |
| **CVC Removal** |  |
| No | 49 (59.7) |
| Yes | 32 (39) |

**Abbreviations:** BSI, bloodstream infection; HD, hemodialysis; CVC, central venous catheter; AVF, arteriovenous fistula; AVG, arteriovenous graft; IQR, interquartile range; SD, standard deviation

**Table S2:** **Laboratory characteristics of HD patients who had BSI**

| **Variable** | **n (%)** |
| --- | --- |
| **Hemoglobin (g/dl), mean ± SD** | 9.84 ± 1.57 |
| **Albumin (g/dl), median and IQR** | 3.6 (3.1-3.91) |
| **Ferritin (ng/ml), median and IQR** | 634 (293-915) |
| **Hepatitis C** |  |
| Negative | 107 (96.4) |
| Positive | 4 (3.6) |
| **Hepatitis B** |  |
| Negative | 110 (99.1) |
| Positive | 1 (0.9) |

**Abbreviations:** BSI, bloodstream infection; HD, hemodialysis; IQR, interquartile range; SD, standard deviation

**Table S3:** **Laboratory characteristics by vascular access type (merged AVF and AVG)**

|  | **CVC** | **AVF/AVG** | **P value (CVC vs. AVF/AVG)** |
| --- | --- | --- | --- |
| **Hemoglobin (g/dl), mean ± SD** | 9.8 ± 1.5 | 9.92 ± 1.75 | 0.751^a^ |
| **Albumin (g/dl), median and IQR** | 3.7 (3.13-3.92) | 3.5 (3-3.7) | 0.054^b^ |
| **Ferritin (ng/ml), median and IQR** | 700.5 (342.57-960.75) | 506.45 (290.75-796.7) | 0.114 ^b^ |

**Abbreviations:** CVC, central venous catheter; AVF, arteriovenous fistula; AVG, arteriovenous graft; IQR, interquartile range; SD, standard deviation

**^a^ Independent sample t-test**

**^b^ Mann-Whitney test**

**Table S4:** **Microbial isolates by vascular access (merged AVF and AVG)**

|  | **CVC** | **AVF/AVG** |
| --- | --- | --- |
| **Microorganism** | **n (%)** | **n (%)** |
| **Gram-negative, total** | **11 (12.3)** | **8 (25.8)** |
| *E. coli non ESBL* | 1 (1.2) | 2 (6.5) |
| *E. coli ESBL* | 1 (1.2) | 1 (3.2) |
| *Klebsiella pneumoniae* | 1 (1.2) | 1 (3.2) |
| *Pseudomonas aeruginosa* | 1 (1.2) | 0 |
| *Stenotrophomonas maltophilia* | 3 (3.5) | 2 (6.5) |
| *Rosemonas gilardii* | 1 (1.2) | 0 |
| *Enterobacter cloacae* | 2 (2.3) | 1 (3.2) |
| *Cedecea lapagei* | 1 (1.2) | 1 (3.2) |
| **Gram-positive, total** | **75 (87.2)** | **23 (74.2)** |
| *CoNS* | 65 (75.6) | 22 (71) |
| *Staphylococcus aureus* | 5 (5.8) | 0 |
| *MRSA* | 1 (1.2) | 0 |
| *Enterococcus faecalis* | 1 (1.2) | 0 |
| *Streptococcus salivarius* | 1 (1.2) | 0 |
| *Bacillus spp.* | 2 (2.3) | 1 (3.2) |
| **Total (117)** | **86 (100)** | **31 (100)** |

**Abbreviations:** CVC, central venous catheter; AVF, arteriovenous fistula; AVG, arteriovenous graft; ESBL, extended spectrum beta-lactamase; CoNS, coagulase-negative staphylococci; MRSA, methicillin-sensitive *Staphylococcus aureus*

Note: the total number of infections shown here is 117 rather than 118 because one infection with CoNS had an unknown access type

**Table S5:** **Rates of antibiotic resistance among the most common gram-positive bacterial isolates**

| **Bacteria** | **Meropenem** | **Ceftazidime** | **Piperacillin**  **/Tazobactam** | **Doxycycline** | **Linezolid** | **Rifampicin** | **Tigecycline** | **Tetracycline** | **Vancomycin** | **Amikacin** | **Gentamicin** | **TMP/SMX** | **Levofloxacin** | **Moxifloxacin** | **Ciprofloxacin** | **Quinupristin-**  **dalfopristin** | **Erythromycin** | **Clindamycin** | **Ceftriaxone** | **Cefuroxime** | **Oxacillin** | **Benzylpenicillin** | **Amoxicillin/ clavulanic acid** |
| --- | --- | --- | --- | --- | --- | --- | --- | --- | --- | --- | --- | --- | --- | --- | --- | --- | --- | --- | --- | --- | --- | --- | --- |
| **Gram-positive bacteria**  **total =99** | **Resistance rate (%)** | | | | | | | | | | | | | | | | | | | | | | |
| ***Staphylococcus aureus* (5)** | - | - | - | - | 0 | 0 | 0 | 0 | 0 | - | 0 | 0 | 0 | 0 | 0 | 0 | 80 | 80 | - | 0 | 0 | 100 | 0 |
| **MRSA (1)** | 0 | 0 | 0 | 0 | 0 | 0 | 0 | 0 | 100 | - | 0 | 0 | 0 | 0 | 0 | 0 | 0 | 0 | - | 100 | 100 | 100 | 100 |
| **[Coagulase-Negative Staphylococci (88)](https://cmr.asm.org/content/27/4/870)** | - | - | 0 | 0 | 0 | 4.5 | 0 | 18 | 0 | - | 35 | 36 | 69 | 39.7 | 70.4 | 0 | 77 | 42.5 | - | 89.5 | 88.6 | 96.5 | 88.5 |
| ***Bacillus* spp. (3)** | 50 | 66.6 | 50 | - | - | - | - | 0 | - | 33 | 0 | - | - | - | 0 | - | 0 | 100 | 100 | 0 | - | - | 66.6 |
| ***Streptococcus salivarius* (1)** | - | - | - | - | 0 | - | 0 | 0 | - | - | - | - | 0 | 0 | - | - | 0 | 0 | 0 | - | - | - | - |
| ***Enterococcus faecalis* (1)** | - | - | - | - | 0 | - | 0 | 0 | - | - | 100 | - | - | - | 100 | 100 | 100 |  | - | - | 0 | 0 | 0 |

**Abbreviations:** -, not tested; TMP/SMX, trimethoprim-sulfamethoxazole; MRSA, methicillin-sensitive *Staphylococcus aureus*

**Table S6: Rates of antibiotic resistance among the most common gram-negative bacterial isolates**

| **Bacteria** | **Imipenem** | **Ertapenem** | **Meropenem** | **Ceftazidime** | **Piperacillin**  **/Tazobactam** | **Cefotaxime** | **Linezolid** | **Tetracycline** | **Amikacin** | **Gentamicin** | **TMP/SMX** | **Levofloxacin** | **Moxifloxacin** | **Ciprofloxacin** | **Ceftriaxone** | **Cefuroxime** | **Cefepime** | **Amoxicillin/ clavulanic acid** | **Ampicillin** |
| --- | --- | --- | --- | --- | --- | --- | --- | --- | --- | --- | --- | --- | --- | --- | --- | --- | --- | --- | --- |
| **Gram negative total (19)** | **Resistance rate (%)** | | | | | | | | | | | | | | | | | | |
| ***E. coli* non ESBL (3)** | - | - | 0 | 50 | 0 | 66.6 | - | - | 0 | - | 50 | - | 0 | - | 0 | - | 66.6 | 33.3 | 0 |
| ***E. coli* ESBL (2)** | 0 | 0 | 0 | 100 | 0 | 100 | 0 | - | 0 | 50 | 100 | - | - | 100 | 100 | 100 | - | 50 | 100 |
| ***Klebsiella pneumonia* (2)** | - | - | 0 | 50 | 0 | 50 | - | - | 0 | 100 | 100 | - | - | 100 | 50 | - | - | 100 | 100 |
| ***Enterobacter cloacae* (3)** | - | 0 | 0 | 0 | - | 0 | - | - | 0 | 0 | - | - | - | 0 | - | - | 0 | 100 | - |
| ***Stenotrophomonas maltophilia* (5)** | - | - |  | 75 | - | - | - | - | - | - | 20 | - | - | - | - | - | 0 | - | - |
| ***Pseudomonas aeruginosa* (1)** | 100 | - | 0 | 0 | - | - | 0 | - | - | 0 | - | - | - | - | - | - | 0 | - | - |
| ***Rosemonas gilardi* (1)** | 0 | 0 | 0 | 100 | 0 | 100 | 0 | - | 0 | 0 | 100 | 0 | - | 0 | - | - | - | 0 | 0 |
| ***Cedecea lapagei* (2)** | 0 | 0 | 0 | 0 | 0 | 0 | 0 | 100 | 0 | 0 | 0 | 0 | 0 | 0 | 0 | - | 0 | 0 | 0 |

**Abbreviations:** -, not tested; TMP/SMX, trimethoprim-sulfamethoxazole; ESBL, extended spectrum beta-lactamase
